# Insights from Vitamin D levels among psoriasis patients in India

**DOI:** 10.6026/973206300210282

**Published:** 2025-03-31

**Authors:** Kapil Raghuwanshi, Anand Saraswat, Sudhakar Petkar, Mahendra Gandhe

**Affiliations:** 1 Department of Biochemistry, C.I.M.S. Chhindwara, Madhya Pradesh, India; 2 Department of Dermatology & Venereology, C.I.M.S. Chhindwara, Madhya Pradesh, India

**Keywords:** Psoriasis, vitamin D level, patients

## Abstract

Psoriasis is a chronic inflammatory skin condition caused by immune system dysfunction characterized by excessive growth of epidermal keratinocytes and inflammatory cells in the dermis and epidermis. The epidermis plays a role in vitamin D synthesis through sunlight exposure. A link between low vitamin D levels and psoriasis is known. Therefore, it is of interest to assess Vitamin D among psoriasis patients to gain insights. Data shows that patients suffering from moderate to severe psoriasis exhibit lower levels of vitamin D when compared to those in the control group. Vitamin D influences keratinocyte proliferation and maturation and hence its role in the management of the condition is realised. However, the effective use of dietary vitamin D or oral supplements for psoriasis treatment remains an unresolved clinical issue with on-going debate regarding the evidence of its benefits.

## Background:

Psoriasis is a chronic inflammatory skin disorder that mainly involves the skin, nails and joints [1]. It is characterized by uncontrolled proliferation of keratinocytes and dysfunctional differentiation due to T cell-mediated dysregulation of immune system [2]. Histologically, the skin lesions in psoriasis are characterized by hyper-proliferation of keratinocytes, impaired epidermal barrier function at the site of skin lesions and skin infiltration by inflammatory cells [3]. Plaque psoriasis, guttate psoriasis, pustular psoriasis, inverse psoriasis, erythro-dermic psoriasis is the different types of psoriasis. Out of all these, Plaque psoriasis is the most common type of psoriasis. Patches of skin become scaly, inflamed and often found on the scalp, elbows or knees. The prevalence of Psoriasis is about 2-3% [1-2]. Although the disease has higher prevalence in the polar regions of the world, its burden in India cannot be underestimated. In India, the prevalence of psoriasis may differ from region to region due to environmental variability and genetic factors. Till date the exact cause of psoriasis has not been known. Several factors contribute to its development, such as auto-immunological, genetic and hormonal factors [4]. The epidermis is the natural source of vitamin D synthesis by the action of UVB of the sun rays. Vitamin D acts as an immuno-modulator and regulates the proliferation and differentiation of skin cells. It has antioxidant and immuno-modulatory properties [3]. Several researches have reported that psoriasis, vitiligo and similar skin disorders have been linked to vitamin D deficiency [4]. Presence of Vitamin D receptors in human epidermal cells and modulation of lipid and cytokine production by it, suggests the possible association between vitamin D and psoriasis pathophysiology. Therefore, it is of interest to assess the Vitamin D levels among psoriasis patients to gain insights for its management.

## Methodology:

The study was done by the Department of Clinical Biochemistry and Department of Dermatology C.I.M.S. Chhindwara (Madhya Pradesh) after approval from institutional ethical committee. 50 clinically diagnosed cases of psoriasis with age group 18-55 years attending C.I.M.S. dermatology OPD in District Hospital Chhindwara (Madhya Pradesh) and 50 healthy individuals of similar age, gender and demographic profile were considered as control. Demographic characters like age, height and weight were recorded and their BMIs were calculated. Age of onset and duration of disease were also recorded. The severity of psoriasis was assessed according to Psoriasis Area and Severity Index (PASI) Score [5]. The PASI is a widely used instrument in psoriasis trials that assess and grades the severity of psoriatic lesions and patients response to treatment. It produces a numeric score ranging from 0 to 72. In general, a PASI score of 5 to 10 is considered moderate disease and a score over 10 is considered severe. In calculating the PASI, severity is determined by dividing the body into four regions: head, upper extremities, trunk and lower extremities, that account for 10%, 20%, 30% and 40% of the total body surface area respectively [6]. Each of these areas is assessed separately for erythema, induration and scaling, which is rated on a scale of 0 (none), 1 (mild symptoms), 2 (moderate symptoms), 3 (severe symptoms), 4 (very severe). After overnight fasting 5 ml of venous blood sample is collected with all aseptic precautions and biochemical analysis is performed, measurement of Serum Vitamin D is done by Enzyme Immunoassay on Thermo-fisher ELISA reader in clinical biochemistry laboratory at C.I.M.S. Chhindwara (Madhya Pradesh). Statistical analysis was performed using the IBM SPSS software version 15 and Microsoft excel sheet.

## Results:

Patient with psoriasis were young and female preponderance was observed as compared with male patients, mean age of onset in case group was 20.3 ± 13.7 years. Majority of patients have lesions on elbows & knees. Mean serum Vitamin D in control group was 27.70±20.95 µg/ml. In the case group, it was 19.13±19.97 µg/ml. The difference was found to be statistically significant (P<0.005), showing a higher serum vitamin D level in control group in comparison to the case group as shown in Figure 1 - (see PDF). Mean vitamin D in PASI stage 1 with mild psoriasis was 30.27±35.79 µg/ml, in stage 2 with moderate psoriasis it was 17.12 ± 6.17 µg/ml and in stage 3 with severe psoriasis it was 12.43±8.45 µg/ml. There was a statistically significant difference seen across the PASI grades (P= 0.047). The lowest mean vitamin D level was seen in PASI stage 3 with severe psoriasis, while it was highest in stage 1 with mild psoriasis.

## Discussion:

Data shows that serum Vitamin D levels in patients with moderate to severe psoriasis are significantly lower compared to those in the control group (p value < 0.05). These findings align with multiple studies that have shown a correlation between lower serum Vitamin D levels and severe psoriasis. Supporting the conclusions of our study, Angela *et al.* [7] in their study also found reduced vitamin D levels in psoriatic patients, compared to healthy controls. Kuang *et al.* [8] demonstrated that risk of psoriasis increased significantly when vitamin D level decreased from 20 to 10 nmol/l. Lisa *et al.* [9] in their study concluded that vitamin D plays a role in metabolic syndrome and improves psoriatic skin lesions. Elrashidy *et al.* [10] found decreased serum vitamin D level in psoriatic patients and stated serum vitamin D level may be used as a marker of psoriasis severity and response to treatment. Contrary to the findings of our study, Mayara *et al.* [11] indicated that serum vitamin D levels did not show a correlation with disease activity in patients with psoriasis. However as per the findings of Barrea *et al.* [12] significant association between low vitamin D and psoriasis have been observed. Vitamin D has supplementation has shown improved outcomes in patients with scalp psoriasis [13]. Vitamin D deficiency has been identified as being linked to psoriasis regardless of factors such as gender, age, smoking habits, family history [14-15].

## Conclusion:

Patients suffering from moderate to severe psoriasis exhibit lower levels of vitamin D when compared to those in the control group. However, additional studies are necessary to establish the connection between vitamin D and psoriasis.

## Funding:

Nil
